# Comparison five primer sets from different genome region of COVID-19 for detection of virus infection by conventional RT-PCR

**Published:** 2020-06

**Authors:** Hamid Reza Mollaei, Abass Aghaei Afshar, Davood Kalantar-Neyestanaki, Mehdi Fazlalipour, Behnaz Aflatoonian

**Affiliations:** 1 Tropical and Infectious Diseases Research Center, Kerman University of Medical Sciences, Kerman, Iran; 2 Medical Mycology and Bacteriology Research Center, Kerman University of Medical Sciences, Kerman, Iran; 3 Department of Arbovirus and Viral Hemorrhagic Fevers (National Ref Lab), Pasteur Institute of Iran (IPI), Tehran, Iran

**Keywords:** Sars-CoV-2, COVID-19, Coronavirus, Reverse transcription-polymerase chain reaction, Specific primer

## Abstract

**Background and Objectives:**

The new beta-coronavirus, which caused Severe Acute Respiratory Coronavirus-2 Syndrome (SARS-CoV-2), a major respiratory outbreak in Wuhan, China in December 2019, is now prevalent in many countries around the world. Identifying PCR-based viruses is a well-known and relatively stable protocol. Unfortunately, the high mutation rates may lead to widespread changes in viral nucleic acid sequences, and so using specific primers for PCR can be recommended. In this study, we evaluated the power of a conventional RT-PCR to detect SARS-CoV-2 RNA among the five set primer sets.

**Materials and Methods:**

The five genomic regions of the Coronavirus SARS-2 virus including Nucleocapsids (N), Envelope (E), RNA depended RNA Polymerase (RdRp), ORF1ab and Spike (S) were selected for primer designing. A conventional RT-PCR was performed to compare sensitivity, specificity and other analytical characteristics of primers designed against two Real Time PCR commercial kits.

**Results:**

The result of the comparative analysis showed that the ORF1ab, N and RdRp primers had a sensitivity, specificity and positive predictive value higher than other primers. A significant difference in the analytical sensitivity between the studied primer sets in RT-PCR kits was observed.

**Conclusion:**

In this study, the ORF1ab, Nucleocapsid and RdRp regions have the best primers for identifying the SARS-CoV-2 RNA between different genes that have been suggested.

## INTRODUCTION

Coronaviruses have a single-stranded positive RNA genome with 26 to 32 kb length. Several animals including camels, bats, mice, dogs and cats have been identified as the hosts of the virus. The sequence of open reading frame (ORF) is common to all beta-corona viruses. ORF1ab encodes most of the enzymatic proteins, and also other proteins such as surface glycoprotein (S), small envelope proteins (E), matrix proteins (M) and nucleocapsid proteins (N) as well as several non-structural proteins are encoded by the virus (1). New mammalian coronaviruses have been identified. For example, a bat-related HKU2-related coronavirus was responsible for an acute fatal diarrhea syndrome in pigs in 2018 (2, 3). The majority of pathogenic coronaviruses in human cause mild symptoms or they are clinically asymptomatic.

In December 2019, a new coronavirus (nCoV-19) has been reported causing outbreaks and infections in humans. The new coronavirus temporarily referred to Wuhan city of China and later renamed to novel Coronavirus (2019-nCoV) or COVID-19 (4). Existing data currently indicate that COVID-19 infects the human population from the bat reservoir, although it is not yet clear whether a currently unknown animal species acts as an intermediate host between the bats and humans (5). Many patients with pneumonia were linked to the Huanan seafood market in Wuhan, Hubei Province, China, where a number of animals, such as birds, bats and rabbits, were already on sale (6). The disease has been spreading rapidly, by epidemic condition in China and then been reported worldwide outbreaks. In February 2020, the World Health Organization (WHO) identified COVID-19, which stands for Coronavirus-2019 (7, 8). For the safety reasons, suspected or documented COVID-19 patient samples should not be used for culture. Therefore, the use of molecular method such as RT-PCR is an important method to identify cases of infection with this virus. Currently, four important regions of the virus have been suggested for molecular identification, including ORF1 (a, b), glycoprotein Spike (E), nucleocapsid (N) and viral RNA polymerase enzyme (RdRp) sequences as the final target of RT-PCR test. Due to the emergence of this virus, identification of infected and suspected cases of COVID-19 is important (4, 9). In this study, we designed and manufactured a best target gene for the detection of nCOVID-19 infection in both qualitative RT-PCR assays.

## MATERIALS AND METHODS

### Sampling.

At the time of the epidemic new Corona virus (COVID-19) in Iran, with the efforts of Kerman University of Medical Sciences, the Kerman Reference Laboratory began to perform Real Time PCR tests for the detection of cases that were diagnosed with nCOVID-19 infection. Therefore, from all of the nasopharynx swab samples over 30 days, 30 viral RNA genomes from the positive samples group and 15 extracted solutions from the negative samples were obtained from Kerman Reference Laboratory. All samples were tested by Real Time PCR method using the kits supplied by TIB (TIB MOLBIOL, Germany) and Sansure (Sansure Biotech, China).

### Ethical clearance.

All clinical samples were obtained during the medical treatment of patients with acute respiratory infection with coronavirus (COVID-19). All patients provided written informed consent, which has been kept on file at the laboratory. Human rights were respected and all ethical issues were considered during this study. The study was supported by the Research Center of Tropical and Infectious Diseases with Reg. No. 98001244 and approved by ethical committee of Kerman University of Medical Sciences (Ethic approval Code: IR.KMU. REC.1398.728).

### Primer designing.

**Fig. 1.**
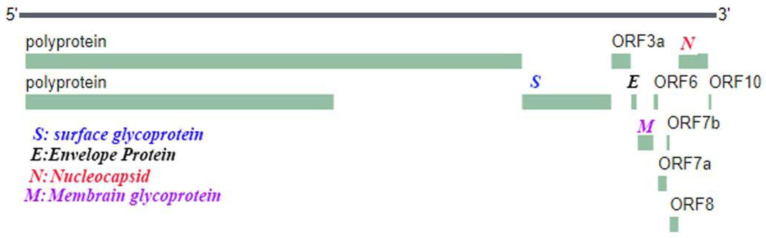
Genome structure of SARS-2 (nCOVID-19) Gene N: Nucleocapsid phosphoprotein: 28274...29533, Gene E: Envelope: 26245...26472 Gene S: Spike: 21563...25384, ORF1ab:266…13468, 13468...21555, Gene M: Membrane glycoprotein: 26523...27191, RdRP:4393…5394

The primer sets were classified into four groups based on specific gene sequences (ORF1, N, E and RdRp) of SARS-2 viruses in the Pubmed. The sequence codes of SARS viruses that used for primer design were: LC528233, MT093631, MT093571, MT084071, MT072688, MT066176, LC528232, LC522973, LC522972, MT027064, MT027062, MT020781, MT123293, MT019530, MT019529, LR757998, LR757997, LR757996, MT123292, LR757995, MT007544, MN996530, MN996528, MN997409, MT123291, MN994467, MN988713, NC045512, MK211378, MK211374, MT123290, MT118835, MT111896, MT106054, MT106053, MT106052, MT159719, MT093631, MT159721, MT020781, MT159718, MT159714, MT020781 and MT192759. The genome of the coronavirus is approximately 29903 ntd (nucleotides) in length and the location of the each gene targeted as follows. Multiple Alignments (ClustalW) was done for finding the conserved nucleotides on all of SARS-2 sequences in EBI –EMBL site (https://www.ebi.ac.uk/Tools/msa/clustalo/). Primers were designed after determination of the reaction conditions such as GC%, Melting temperature (Tm), the length of the primer and the product range based on online tools (https://www.eurofinsgenomics.eu/en/ecom/tools/pcr-primer-design/). Sequences of all the primers and their respective locations within the SARS-CoV-2 genome which was used for the in-house assays are shown in Fig. 1.

### One Step RT-PCR.

The RT-PCR assay was performed using the One-Step supermix RT-PCR Master Mix (Biosystem, Germany). Each 25-μL reaction mixture contained 12.5 μL of 2× Master Mix, 1 μL of the 20× RNase Inhibitor mix, 0.25 μL of 50 μM forward and 0.75 μL of 50 μM reverse primers, 6.125 μL of nuclease-free water, and 5 μL of nucleic acid extract. The amplification was carried out in on a MWG Thermo cycler System (MWG, Germany). The thermo cycling conditions consisted of 30 min at 50°C for reverse transcription, 5 min at 95°C for activation of the Taq DNA polymerase, and 45 cycles of 15 s at 95°C, 10 s in 58°C and 40 s at 60°C.

### Detection of RT-PCR product.

The PCR products were electrophoresis in 1.5% agarose gel and were stained with Red safe. The gels were visualized under UV light and Vilberlumert gel documentation.

### Specificity and sensitivity of primers.

RT-PCR test was done for different viral genomes for each primer sets such as Influenza virus (A, H1N1, H3N2, B), Para influenza virus 1,3, Hepatitis C virus (HCV), Human Herpes simplex virus (HSV1,2), Human Immunodeficiency virus (HIV-1), Corona-OC43, HCoV-NL63, MERS-CoV, Rhinovirus/ Enterovirus, Respiratory syncytial virus (A/B) and Human metapneumovirus. For sensitivity, we prepared a ten-fold serial dilution of the mixture from nCOVID-19 RNAs positive. That level of RNA mixture was determined by a commercial kit. (Genesig, Primerdesign Ltd). There was 10^6^ copies/ml.

## RESULTS

### Gel electrophoresis.

**Fig. 2.**
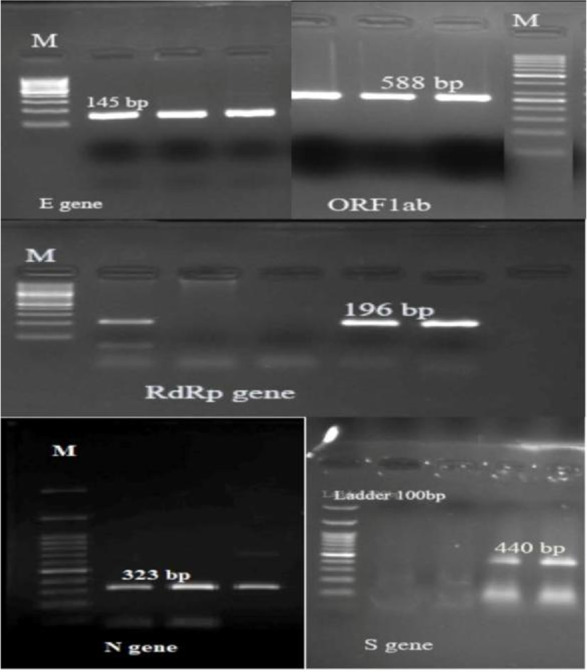
Gel electrophoresis of five products in 2% gel agarose, M: Marker 100 bp E gene; 145 bp, ORF1ab: 588 bp, RdRp: 196 bp, N gene: 323 bp, S gene: 440 bp

The sizes of products for each primer for the in-house RT-PCR protocol are shown in Table 1 and 2 respectively. The amplicon generated by the specific primers has different molecular weight; therefore, the products could be differentiated using agarose gel electrophoresis (Fig. 2). Multiple bands were not produced from patients with nCOVID-19 disease (Table 2).

**Table 1.** The sequences of specific primers for detection of COVID-19

**Target gene**	**Forward/ Reverse primer**	**%GC**	**Tm°C**	**Location**	**Product size**
ORF1ab	CTAGGACCTCTTTCTGCTCA ACACTCTCCTAGCACCATCA	50/50	60.5/60.5	10536–11124	588 bp
S	CCCTGTTGCTATTCATGCAG CCCTATTAAACAGCCTGCAC	50/45	60.1/59.8	1859–1955	440 bp
E	GGAAGAGACAGGTACGTTAA AAGGTTTTACAAGACTCACG	45/40	61.1/60.6	17–162	145 bp
N	CCTCTTCTCGTTCCTCATCA CCTGGTCCCCAAAATTTCCT	50/50	61.3/62.3	544–867	323 bp
RdRp	CATCTCACTTGCTGGTTCCT CCTTAATAGTCCTCACTTCTCTC	50/43.5	60.8/60.3	384–580	196 bp

**Table 2.** RT-PCR products generated in this study

**Gene**	**PCR product generated in RT-PCR**
RdRp	CATCTCA CTTGCTGGTT 400 CCTATAAAGA TTGGTCCTAT TCTGGACAAT CTACACAACT AGGTATAGAA 450 TTTCTTAAGA GAGGTGATAA AAGTGTATAT TACACTAGTA ATCCTACCAC 500 ATTCCACCTA GATGGTGAAG TTATCACCTT TGACAATCTT AAGACACTTC 550 TTTCTTTGAG AGAAGTGAGG ACTATTAAGG Number of CpGs in the PCR product: 0
ORF1ab	CTAGG ACCTCTTTCT 10550 GCTCAAACTG GAATTGCCGT TTTAGATATG TGTGCTTCAT TAAAAGAATT 10600 ACTGCAAAAT GGTATGAATG GACGTACCAT ATTGGGTAGT GCTTTATTAG 10650 AAGATGAATT TACACCTTTT GATGTTGTTA GACAATGCTC AGGTGTTACT 10700 TTCCAAAGTG CAGTGAAAAG AACAATCAAG GGTACACACC ACTGGTTGTT 10750 ACTCACAATT TTGACTTCAC TTTTAGTTTT AGTCCAGAGT ACTCAATGGT 10800 CTTTGTTCTT TTTTTTGTAT GAAAATGCCT TTTTACCTTT TGCTATGGGT 10850 ATTATTGCTA TGTCTGCTTT TGCAATGATG TTTGTCAAAC ATAAGCATGC 10900 ATTTCTCTGT TTGTTTTTGT TACCTTCTCT TGCCACTGTA GCTTATTTTA 10950 ATATGGTCTA TATGCCTGCT AGTTGGGTGA TGCGTATTAT GACATGGTTG 11000 GATATGGTTG ATACTAGTTT GTCTGGTTTT AAGCTAAAAG ACTGTGTTAT 11050 GTATGCATCA GCTGTAGTGT TACTAATCCT TATGACAGCA AGAACTGTGT 11100 ATGATGATGG TGCTAGGAGA GTGT Number of CpGs in the PCR product: 3
S	TGCTG ACACTACTGA TGCTGTCCGT GATCCACAGA CACTTGAGAT 1750 TCTTGACATT ACACCATGTT CTTTTGGTGG TGTCAGTGTT ATAACACCAG 1800 GAACAAATAC TTCTAACCAG GTTGCTGTTC TTTATCAGGA TGTTAACTGC 1850 ACAGAAGTCC CTGTTGCTAT TCATGCAGAT CAACTTACTC CTACTTGGCG 1900 TGTTTATTCT ACAGGTTCTA ATGTTTTTCA AACACGTGCA GGCTGTTTAA 1950 TAGGGGCTGA ACATGTCAAC AACTCATATG AGTGTGACAT ACCCATTGGT 2000 GCAGGTATAT GCGCTAGTTA TCAGACTCAG ACTAATTCTC CTCGGCGGGC 2050 ACGTAGTGTA GCTAGTCAAT CCATCATTGC CTACACTATG TCACTTGGTG 2100 CAGAAAATTC AGTTGCTTAC TCTAATAACT CTATTGCCAT ACCCAC Number of CpGs in the PCR product: 7
N	CCTCTTC 550 TCGTTCCTCA TCACGTAGTC GCAACAGTTC AAGAAATTCA ACTCCAGGCA 600 GCAGTAGGGG AACTTCTCCT GCTAGAATGG CTGGCAATGG CGGTGATGCT 650 GCTCTTGCTT TGCTGCTGCT TGACAGATTG AACCAGCTTG AGAGCAAAAT 700 GTCTGGTAAA GGCCAACAAC AACAAGGCCA AACTGTCACT AAGAAATCTG 750 CTGCTGAGGC TTCTAAGAAG CCTCGGCAAA AACGTACTGC CACTAAAGCA 800 TACAATGTAA CACAAGCTTT CGGCAGACGT GGTCCAGAAC AAACCCAAGG 850 AAATTTTGGG GACCAGG Number of CpGs in the PCR product: 6
E	GGAA GAGACAGGTA CGTTAATAGT TAATAGCGTA 50 CTTCTTTTTC TTGCTTTCGT GGTATTCTTG CTAGTTACAC TAGCCATCCT 100 TACTGCGCTT CGATTGTGTG CGTACTGCTG CAATATTGTT AACGTGAGTC 150 TTGTAAAACC TT Number of CpGs in the PCR product: 5

CpG: Number of CpG islands in the unmodified PCR product

### Specificity of primers.

No positive result was seen with non–SARS-CoV2 RNA from the specificity panel. However, when PCR products were subjected to agarose gels electrophoresis analysis, in S and E primer sets in some samples multiple bands were present, which indicated the nonspecific products (data not shown). They may have mutated.

No cross-reactivity was observed when primers for E and N genes were used together in multiplex RT-PCR against COVID-19 virus. RNA from SARS-CoV2 was also tested using all the primers described above, and no cross-reactivity was observed (data not shown).

### Commercial Real Time PCR and in-house conventional RT-PCR results.

The results of the RT-PCR test on 30 infected patients with COVID-19 were shown in Table 2. The COVID-19 RNA was detected from 22 samples (73.3%) of total samples from RdRp gene, 83.3% (25 samples) for Nucleocapsid (N) and S (surface protein) genes, 60% (18 samples) for E gene and 80% (24 samples) for ORF1ab region.

### Specification of sunsure Biotech Real Time PCR kit.

Viral RNA is used for detection of three regions RdRp and N genes of novel coronavirus (2019-nCoV) in respiratory secretion of suspected patients with novel coronavirus infection. The RdRp region was detected in FAM, ROX for N and HEX channel to test internal control. The test is positive if there is typical S-shape amplification curve at FAM, ROX and/or Hex channel, and the amplification curve must be detected at Ct ≤ 35. If there is no typical S-shape amplification with no Ct or Ct > 40, the result is negative. The limit of detection of this kit is 200 copies/mL.

### Specification of TIB MOLBIOL Real Time PCR kit.

Viral RNA is used for qualitative detection of the ORF1ab, E and N genes of novel coronavirus (2019-nCoV) in nasopharyngeal swab, oropharyn-geal swab, alveolar lavage fluid, sputum, serum, whole blood and feces from suspected pneumonia cases with novel coronavirus infection. ORF-1ab, E and N region was detected in 530 channel (FAM) and 660 channel (CY5) to test internal control. The test is positive if there is typical S-shape amplification curve and the amplification curve detected at Ct ≤ 40. If there is no typical S-shape amplification with no Ct or Ct > 40, the result is negative. It is indicated that the specimen’s concentration is too low, or there are interfering substances that inhibit the reaction and test result is invalid. The limit of detection of this kit is 10 copies/mL.

### Sensitivity and specificity of different primers.

The sensitivity (also called true positive rate, recall or the probability of disease in detection with the certain tests) measures the proportion of true positive that is correctly identified as such (e.g., percentage of patients which are correctly identified by these methods). Specificity (also called the real negative rate) measures the real negatives that are correctly identified as such (e.g., the percentage of healthy people who have a negative test). In this study, the TIB MOLBIOL Kit and Sansure Biotech Kit are considered as reference methods and were compared with our designed in-house primers for detection of COVID-19. The results of this comparison and analysis are presented in Tables 3 and 4.

**Table 3.** Comparison of commercial kits and conventional RT-PCR results indifferent Genes.

**Sample No.**	**A: TIB MOLBIOL Kit**			**B: Sansure Biotech kit**		**In-House Primers**				

	**N gene**	**RdRp gene**	**E gene**	**N gene**	**RdRp gene**	**RdRp gene**	**N gene**	**E gene**	**S gene**	**ORF1ab gene**
1	P	P	P	P	P	P	P	P	P	P
2	P	P	N	P	N	P	P	N	P	N
3	P	P	P	P	P	P	P	P	P	P
4	P	P	P	P	P	P	P	P	P	P
5	P	N	N	P	N	N	P	N	N	N
6	P	P	N	P	P	P	P	P	P	P
7	P	P	P	P	N	P	N	P	N	P
8	P	P	N	P	P	P	P	N	P	P
9	N	P	N	N	P	P	N	N	P	P
10	P	N	N	P	N	N	P	N	P	N
11	P	P	P	P	P	P	P	P	P	P
12	P	P	N	P	P	P	P	N	P	P
13	P	N	N	P	P	N	P	N	N	P
14	P	P	N	P	P	P	P	P	P	P
15	P	P	N	P	N	P	P	P	N	N
16	P	N	P	P	P	N	P	P	P	P
17	P	P	N	P	P	P	P	P	N	P
18	N	P	P	N	P	P	N	N	P	P
19	P	N	P	P	P	N	N	P	P	N
20	P	P	N	P	P	P	P	P	P	P
21	P	P	P	P	P	P	P	P	N	P
22	N	P	P	P	P	P	N	N	P	P
23	P	N	P	P	N	P	P	P	N	N
24	P	P	N	P	P	P	P	P	P	P
25	P	P	N	P	P	P	P	N	P	P
26	N	P	P	P	P	N	P	P	P	P
27	P	N	P	P	P	N	P	P	N	P
28	P	P	N	P	P	P	P	N	P	P
29	P	N	P	P	P	N	P	P	N	P
30	N	P	P	P	P	P	P	N	P	P
Total P/N	24/6	22/8	15/15	28/2	24/6	22/8	25/5	18/12	21/9	24/6

A: TIB MOLBIOL COVID-19 kits: Monoplex real time PCR for E, N, RdRp gene B: Sansure Biotech COVID-19 kit: Multiplex real time PCR for N (Nucleocapsid), RdRp (RNA depended RNA Polymerase), E (Spike glycoprotein), S (Surface protein) gene, P: Positive, N: Negative

**Table 4.** Comparison results of in-house RT-PCR by new designed primers

**Analysis tests**	**Target Genes**				

	**RdRp**	**N**	**E**	**S**	**ORF1ab**
% Sensitivity	95.7	96.6	66.7	85.7	96.0
% Specificity	88.9	100.0	66.7	50.0	100.0
% Positive Predictive Value (PPV)	95.7	100.0	88.9	96.0	100.0
% Negative Predictive Value (NPV)	88.9	50.0	33.3	20.0	83.3
False Negative Rate(FNR)	4.3	3.4	33.3	14.3	4.0
Fall out, False Positive Rate(FPR)	11.1	0.0	33.3	50.0	0.0
False Discovery Rate (FDR)	4.3	0.0	11.1	4.0	0.0
False omission Rate (FOR)	11.1	50.0	66.7	80.0	16.7
Threat Score, Critical Success Index (CSI)	91.7	96.6	61.5	82.8	96.0
% Accuracy	47.0	31.3	29.3	27.3	40.7

## DISCUSSION

In early response and detection of the SARS-2 (nCOVID-19) outbreak, the cause of severe acute respiratory infection in Iran, we developed a conventional RT-PCR assay based on multiple primer sets designed to different genomic targets to facilitate sensitive and specific detection of SARS-CoV2 in all laboratories. No cross reactivity was observed when PCR products were analyzed. Therefore, by performing and designing this method, it is not necessary to use dangerous, time consuming and expensive methods such as cell culture for diagnosing nCOVID-19, which is impossible in all laboratory and requires Biosafety Level 3 (BSL3) laboratory. The high potential for N gene and RdRp gene in detection of SARS-CoV2 was demonstrated in nucleic acid extraction from respiratory secretions (10). The increased sensitivity of the RT-PCR assay over cell culture and equal to commercial RT-PCR methods may help to detection of nCOVID-19 at earlier stages of infection, when the virus is present at low titer in respiratory secretions (11). For the detection of SARS-Cov2 (nCOVID-19) in patients with respiratory infection, we designed a conventional RT-PCR method from five regions of virus. The results of RT-PCR for N gene were the most positive in commercial kits and the method designed in this study. Therefore, this gene is the most frequent gene in the coronavirus infection, whereas the E gene has the least positive in this infection. As shown in the commercial kit produced by the TIB MOLBIOL (Germany), the E gene results in patients were positive only fifty percent. This gene has not been used in the Sunsure Biotech Kit (China). Therefore, the best genes for identifying cases of the new coronavirus are the N Gene and viral RNA polymerase, respectively. The RNA depended RNA polymerase has overlaps with N-terminal region of ORF1ab (12). Besides the 2 commercial kits described above, five other sets of primers were designed for conventional RT-PCR. Primer sets of ORF1 and RdRP target the different genes and produce their own product while the two regions have overlap in N-terminal ORF1. Although the sensitivity of these two primers to detect coronavirus was equal, the ORF1 primer had a specificity and positive predictive value of 100%. The false negative rate with this primer was only 4%. The percentage of false positive and false discovery rate were zero. So, use of this region for diagnosis of coronavirus is highly recommended (13). The nucleocapsid gene primers (N) had the highest sensitivity among the designed primers (96.6%). The specificity and positive predictive value for these primers was calculated 100%. The probability of obtaining false positive and false discovery rate results with these primers was zero percent. Negative results of this test were valid in only 50% of patients with the disease. Therefore, the possibility of obtaining false negative results can be decreased by using simultaneous primers in the ORF1ab and nucleocapsid (N) region (14). As shown in Table 4, the diagnostic value of the E gene is lower than other genes in the sample because the E gene is low (Fig. 1). The sensitivity and the specificity were 66.7%. The critical success index for E gene was 61%. The surface gene (S) of COVID-19 as a target for detection of coronavirus infection in this study had a sensitivity of 85% and specificity of 50%, and was only able to identify 21 specimens (70%) positively (15). The positive result in the test was probably 95% true and the negative results had the validity of 20%. In commercial kits, two or more pairs of simultaneous primers beside internal control gene were also used in a single reaction (16). Using simultaneous multiple genes in a reaction create a competitive replication state that is depended to decreases or increases each of genes those have effects on other replication (17). It is therefore recommended that these methods may not be used simultaneously and not more than two pairs of primers in one reaction, unless their expression and amplification rate are the same and may not be affected by the others. This is one of the reasons why the results of the E gene are often false negative. Also, the use of multigene reactions cause the minimum detectable range to increase drastically, and low rate of viral loads in cases where the shading of virus is low, are not detectable and results can be false negatives (18). This is one of the cases where these two commercial kits are widely used as clinical signs and other blood tests indicate coronary disease, but their diagnostic tests are false negative (19).

Although real-time RT-PCR test offers clear advantages over conventional RT-PCR, the results still need to be interpreted with caution. For instance, the effectiveness of RT-PCR in the diagnosis of SARS-CoV2 in clinical samples has been shown to be greatly affected by the quantity, type and timing of sample collection. False-negative results can also be a concern due to the poor quality of the nucleic acid or the presence of RT-PCR inhibitors. We expressed this by simultaneously testing the human RNase P gene, which should be present in all suitable collected samples (20, 21). False-negative results could also potentially result from mutations in the primer, probe target regions and in the COVID-19 virus genome. We have made this available by taking into several genetic targets in the assessment and by comparing the primer probe sequences and published sequences of COVID-19 virus (22, 23). To prevent false-positive results, careful testing by avoiding viral RNA that has been already infected or being contaminated with the PCR product during the preparation of genome extracts or PCR reactions must be considered. In addition, all positive RT-PCR samples were tested and confirmed in the second laboratory using Real-time PCR based on different genetic targets (24).

## CONCLUSION

The RT-PCR assay with N, RdRp, ORF1 primers permitted rapid, sensitive, and specific detection for COVID-19 in patients with pneumonia and provided diagnostic support during the recent COVID-19 outbreak in low level laboratory that do not have a Real Time PCR instrument. Widely deploying this assay through the health centers will enhance their ability to provide a rapid response in the event of the possible return of COVID-19.

## References

[1] Lu R Zhao X Li J Niu P Yang B Wu H Genomic characterisation and epidemiology of 2019 novel coronavirus: implications for virus origins and receptor binding. Lancet 2020;395:565–574. 32007145 10.1016/S0140-6736(20)30251-8 PMC7159086

[2] Madhi A Ghalyanchilangeroudi A Soleimani M. Evidence of human coroanvirus (229E), in patients with respiratory infection, Iran, 2015: the first report. Iran J Microbiol 2016;8:316–320. 28149491 PMC5277600

[3] Hu ZB Ci C. [Screening and management of asymptomatic infection of corona virus disease 2019 (COVID-19)]. Zhonghua Yu Fang Yi Xue Za Zhi 2020;54:E025. 10.3760/cma.j.cn112150-20200229-00220 32388946

[4] Paraskevis D Kostaki EG Magiorkinis G Panayiotakopoulos G Sourvinos G Tsiodras S. Full-genome evolutionary analysis of the novel corona virus (2019-nCoV) rejects the hypothesis of emergence as a result of a recent recombination event. Infect Genet Evol 2020;79:104212. 32004758 10.1016/j.meegid.2020.104212 PMC7106301

[5] Rahman Qureshi UU Saleem S Khan A Afzal MS Ali MS Ahmed H. Outbreak of novel Corona virus (2019-nCoV); implications for travelers to Pakistan. Travel Med Infect Dis 2020;101571. doi: 10.1016/j.tmaid.2020.101571. 32032674 PMC7129558

[6] Wu J Wu X Zeng W Guo D Fang Z Chen L Chest CT findings in patients with Corona virus disease 2019 and its relationship with clinical features. Invest Radiol 2020;55:257–261. 32091414 10.1097/RLI.0000000000000670 PMC7147284

[7] Xiong Z Fu L Zhou H Liu JK Wang AM Huang Y [Construction and evaluation of a novel diagnosis process for 2019-Corona Virus Disease]. Zhonghua Yi Xue Za Zhi 2020;100:1223–1229. 32157849 10.3760/cma.j.cn112137-20200228-00499

[8] Xu K Cai H Shen Y Ni Q Chen Y Hu S [Management of corona virus disease-19 (COVID-19): the Zhejiang experience]. Zhejiang Da Xue Xue Bao Yi Xue Ban 2020;49:0. 10.3785/j.issn.1008-9292.2020.02.02 PMC8800711 32391658

[9] Lu R Zou L Wang Y Zhao Y Zhou W Wu J [Sequencing and phylogenetic analyses of structural and accessory proteins of middle east respiratory syndrome Coronavirus from the first imported case in China, 2015]. Bing Du Xue Bao 2015;31:333–340. 26524904

[10] Corman VM Landt O Kaiser M Molenkamp R Meijer A Chu DKW Detection of 2019 novel corona-virus (2019-nCoV) by real-time RT-PCR. Euro Surveill 2020;25:2000045. 10.2807/1560-7917.ES.2020.25.3.2000045 PMC6988269 31992387

[11] To KK Tsang OT Chik-Yan Yip C Chan KH Wu TC Chan JMC Consistent detection of 2019 novel coronavirus in saliva. Clin Infect Dis 2020;ciaa149. doi: 10.1093/cid/ciaa149. PMC7108139 32047895

[12] Wang CB. [Analysis of low positive rate of nucleic acid detection method used for diagnosis of novel coronavirus pneumonia]. Zhonghua Yi Xue Za Zhi 2020;100:961–964. 32077662 10.3760/cma.j.cn112137-20200213-00280

[13] Won J Lee S Park M Kim TY Park MG Choi BY Development of a laboratory-safe and low-cost detection protocol for SARS-CoV-2 of the Coronavirus disease 2019 (COVID-19). Exp Neurobiol 2020;24:326–333. doi: 10.5607/en20009. PMC7237269 32156101

[14] Xie C Jiang L Huang G Pu H Gong B Lin H Comparison of different samples for 2019 novel coronavirus detection by nucleic acid amplification tests. Int J Infect Dis 2020;93:264–267. 32114193 10.1016/j.ijid.2020.02.050 PMC7129110

[15] Zhang R Li JM. [The way to reduce the” false negative results” of 2019 novel coronavirus nucleic acid detection]. Zhonghua Yi Xue Za Zhi 2020;100:E008. 32072795 10.3760/cma.j.issn.0376-2491.2020.0008

[16] Zhang ZW Zhou YM Zhang Y Guo Y Tao SC Li Z Sensitive detection of SARS coronavirus RNA by a novel asymmetric multiplex nested RT-PCR amplification coupled with oligonucleotide microarray hybridization. Methods Mol Med 2005;114:59–78. 16156097 10.1385/1-59259-923-0:59 PMC7122606

[17] Yu CH Liu LT Liu S Feng YY Wang CR Li HL [Enhanced-real time PCR: a highly sensitive method for SARS-coronavirus detection]. Beijing Da Xue Xue Bao Yi Xue Ban 2006;38:211–213. 16617369

[18] Nguyen T Duong Bang D Wolff A. 2019 Novel Coronavirus Disease (COVID-19): Paving the Road for Rapid Detection and Point-of-Care Diagnostics. Micromachines (Basel) 2020;11:306. 10.3390/mi11030306 PMC7142866 32183357

[19] Okba NMA Widjaja I Li W GeurtsvanKessel CH Farag E Al-Hajri M Serologic detection of middle east respiratory syndrome Coronavirus functional antibodies. Emerg Infect Dis 2020;26: 1024–1027. 32150528 10.3201/eid2605.190921 PMC7181916

[20] Peiris JS Poon LL. Detection of SARS coronavirus. Methods Mol Biol 2011;665:369–382. 21116811 10.1007/978-1-60761-817-1_20 PMC7121416

[21] Pfefferle S Reucher S Norz D Lutgehetmann M. Evaluation of a quantitative RT-PCR assay for the detection of the emerging coronavirus SARS-CoV-2 using a high throughput system. Euro Surveill 2020;25:2000152. 10.2807/1560-7917.ES.2020.25.9.2000152 PMC7068162 32156329

[22] Spackman E Kapczynski D Sellers H. Multiplex real-time reverse transcription-polymerase chain reaction for the detection of three viruses associated with poult enteritis complex: turkey astrovirus, turkey coronavirus, and turkey reovirus. Avian Dis 2005;49:86–91. 15839418 10.1637/7265-082304R

[23] Stranieri A Lauzi S Giordano A Paltrinieri S. Reverse transcriptase loop-mediated isothermal amplification for the detection of feline coronavirus. J Virol Methods 2017;243:105–108. 28109842 10.1016/j.jviromet.2017.01.009 PMC7113849

[24] Tang A Tong ZD Wang HL Dai YX Li KF Liu JN Detection of novel Coronavirus by RT-PCR in stool specimen from asymptomatic child, China. Emerg Infect Dis 2020;26:1337–1339. 32150527 10.3201/eid2606.200301 PMC7258461

